# Promoting community health and climate justice co-benefits: insights from a rural and remote island climate planning process

**DOI:** 10.3389/fpubh.2024.1309186

**Published:** 2024-03-12

**Authors:** Angel M. Kennedy, Kiera Tsakonas, Forrest Berman-Hatch, Sophia Conradi, Max Thaysen, Manda Aufochs Gillespie, Maya K. Gislason

**Affiliations:** ^1^Faculty of Health Sciences, Simon Fraser University, Burnaby, BC, Canada; ^2^Women’s Health Research Institute, Vancouver, BC, Canada; ^3^Cortes Island Academy, Cortes Island, BC, Canada; ^4^Anthropology and Political Science, Faculty of Arts, University of British Columbia, Vancouver, BC, Canada; ^5^Cortes Island Community Foundation, Cortes Island, BC, Canada; ^6^Friends of Cortes Island, Cortes Island, BC, Canada; ^7^BC Emergency Health Services, Cortes Island, BC, Canada; ^8^Folk University, Cortes Island, BC, Canada

**Keywords:** climate change, community planning, health, rural, co-benefits, data equity

## Abstract

Climate change is an environmental crisis, a health crisis, a socio-political and an economic crisis that illuminates the ways in which our human-environment relationships are arriving at crucial tipping points. Through these relational axes, social structures, and institutional practices, patterns of inequity are produced, wherein climate change disproportionately impacts several priority populations, including rural and remote communities. To make evidence-based change, it is important that engagements with climate change are informed by data that convey the nuance of various living realities and forms of knowledge; decisions are rooted in the social, structural, and ecological determinants of health; and an intersectional lens informs the research to action cycle. Our team applied theory- and equity-driven conceptualizations of data to our work with the community on Cortes Island—a remote island in the northern end of the Salish Sea in British Columbia, Canada—to aid their climate change adaptation and mitigation planning. This work was completed in five iterative stages which were informed by community-identified needs and preferences, including: An environmental scan, informal scoping interviews, attending a community forum, a scoping review, and co-development of questions for a community survey to guide the development of the Island’s climate change adaptation and mitigation plan. Through this community-led collaboration we learned about the importance of ground truthing data inaccuracies and quantitative data gaps through community consultation; shifting planning focus from deficit to strengths- and asset-based engagement; responding to the needs of the community when working collaboratively across academic and community contexts; and, foregrounding the importance of, and relationship to, place when doing community engagement work. This suite of practices illuminates the integrative solution-oriented thinking needed to address complex and intersecting issues of climate change and community health.

## 1 Introduction

The Intergovernmental Panel for Climate Change (IPCC) states that climate change is producing myriad direct and indirect adverse health impacts, from increased morbidity and mortality rates due to acute trauma and disease emergences and amplification in human and animal populations through to mental health issues arising from significant climate events and their sequalae such as displacement, social and economic losses, malnutrition and food insecurity (1, 2). These effects are disproportionately felt by children, older adults, those who are structurally marginalized and those who live close to the land, such as Indigenous peoples (3–5). With each season where climactic records are broken and climate disasters occur in quick succession, people come to understand that climate change is not a distant future challenge but a present-day reality. For example, in British Columbia (BC), the wildfires, floods and heatwaves experienced in 2021 have shifted climate change narratives from a focus on projected impacts to current and urgent conditions to be addressed (6). The impacts of climate events and community responses to them are diverse and require careful consideration.

It is important that social narratives about climate change are informed by data that reflects the nuances of various ways and forms of knowledge; roots an understanding of issues in the social, structural, and ecological determinants of health; and takes an intersectional lens in understanding how social, political, and economic structures impact people’s diverse experiences. The BC Assembly of First Nations, in their BC First Nations Climate Strategy and Action Plan, for example, highlights both cross cutting and community specific climate risks that Indigenous Peoples experience due to the ongoing impacts of settler colonialism and capitalism. This work identifies that the long term effects of initial and ongoing land dispossession and assimilation policies pursued by the Canadian State since colonization has impacted where Indigenous Peoples live, their socio-economic conditions, and how connection and relationship to Mother Earth is exercised, experienced and respected (7). Broadly, we concur with the importance of rooting climate change responses in data on the social and ecological determinants of health that is equity-informed. We link these data equity commitments to the importance of grounding work in place given the richness of insights gained during the community-led climate change work we conducted on Cortes Island, BC, Canada.

When working on issues of equity, we also emphasize the importance of engaging in reflexive practice and making explicit our positionalities, social locations, biases, and influences as they relate to research, practice, adaptation and mitigation and broadly reflecting on the social and material environments we occupy (8, 9). The co-authors of this article make up a diverse group of activists, community-engaged researchers, and educators, with five out of seven authors growing up on, or currently living on, Cortes Island. Members of our group have spent various years engaging in critical thinking about issues of equity-oriented data science, with disciplinary backgrounds spanning health sciences, public health, education, anthropology, sociology, environmental management, and geography. Our focus on interactions within socio-ecological systems is intended to help us understand the links between social systems and ecosystems (10). In acknowledging how social, economic and ecological systems are connected, we can then work to consider how future changes and adaptations may affect communities. It is through these lenses that we are sharing insights from our collaborative community-informed work on climate change planning on Cortes Island.

## 2 Context

**Figure 1:**
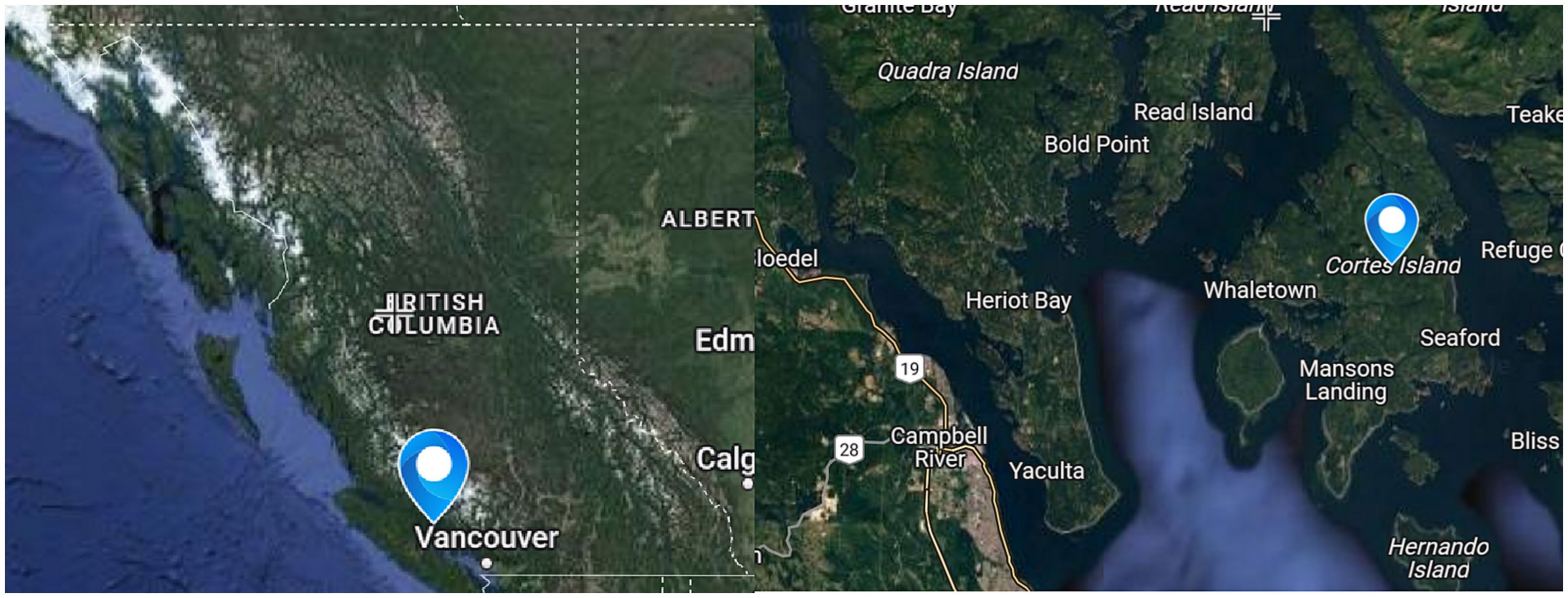
Position of cortes island within british columbia (left) and map of cortes island (right).

Cortes Island is located in the Discovery Islands Archipelago, situated between Vancouver Island and the British Columbia Mainland in the northern reaches of the Salish Sea (see Figure 1). This 25 km long island (130km^2^) is the unceded traditional land of the Coast Salish First Nations, specifically the toq qaymɩxʷ (Klahoose), ʔop qaymɩxʷ (Homalco), and ɬəʔamɛn qaymɩxʷ (Tla’amin) Nations. Cortes Island has roughly 1,000 permanent residents, and a median household income that is approximately $30,000 less than BC’s average (11).

Rural and remote island and coastal communities, such as Cortes Island, are disproportionately impacted by climate change. Cortes Island is accessible only by float plane, boat or ferry, yielding vast carbon emissions due to the transportation of people, goods and services, all of which can be easily disrupted during climate events. Despite unreliable and cost prohibitive access to basic goods and services, islanders have limited access to provincial and federal government services. For example, extreme weather events can impact access to healthcare on Cortes Island as the closest hospital is located two ferry rides away in Campbell River. A low median household income, limited housing stock and a higher cost of healthy foods pose significant challenges for Cortes Island residents who are already contending with myriad intersecting issues such as high rates of child poverty, increasing extreme weather patterns (e.g., droughts and heatwaves) and related climate change impacts to housing and food security (e.g., shellfish impacted by ocean acidification and intertidal species casualties from heatwaves and fires) (11–14). While considerations of intersecting social dimensions of health are often lacking from community planning processes and datasets, in communities like Cortes Island it is crucial to view community conditions through an integrative and holistic lens. Without community input, the development of community plans, policies and processes that are informed purely by quantitative data can be misleading and reduce effective short-, medium- and long-term planning. In response, our work was rooted in the premise that when considering what “effective” climate change adaptation for Cortes Island would look like, solutions must be grounded in community knowledge and perspectives, work at both the individual and community levels, and actively engage with the eco-social contexts within which climate change experiences and responses are rooted.

## 3 Methodology

This project was built through a partnership created between the Research for Eco-Social and Equitable Transformation (RESET) Lab at Simon Fraser University and the Friends of Cortes Island (FOCI) Society with the aim to gather data and formulate a survey to inform the island’s climate mitigation and adaptation planning process. This partnership emerged from a need identified by the community (through FOCI) for mitigation and adaptation planning at the community level. FOCI contacted the PI of the RESET Lab, to support this work. As the PI and several members of the research team either grew up on or currently live on Cortes also, we bring a unique insider/outsider perspective and positionality to our work together (15).

Our work for Cortes Island’s climate planning process took place in five phases from May 2022 to August 2023. Phase 1: This partnered work began with an environmental scan of existing environmental and person-centered data about Cortes Island [see Supplementary material (Datasets Identified in Environmental Scan) for a summary of which datasets were included] (16). Phase 2: The next step of our partnership led to five informal scoping-related interviews with community members about their knowledge and expertise related to the assets on the island that were not represented in the data captured in the environmental scan, such as wetlands and old growth forest ecosystems (17). Throughout this project, we engaged in an integrated knowledge translation (iKT) framework, where outputs were given back to the community throughout the process of the work together (18). For example, during this stage of the project, a drought report, a community asset report, and an information sheet on heat-pumps was developed for the community. These outputs were requested by FOCI and were based off the environmental scan and informal community scoping interviews we conducted. Phase 3: In the third stage of the project, we attended a community planning forum for the social profit sector (19), where we participated in a climate change focus group that the Island was running with six community organizers about mitigation and adaptation to climate change events on Cortes Island. During this gathering it became evident that the community’s climate adaptation planning process would benefit from a synthesis about existing guidance on justice-informed community engagement strategies (20). Phase 4: After this gathering, we conducted a scoping review of peer-reviewed and gray literature asking how recognitional, procedural, and distributive justice can be used as a lens for progressing community engagement in local climate planning processes. Search terms, databases, and PRISMA chart of papers can be found in Supplementary material (PRISMA Chart of Scoping Review and Search Strategy, Terms, and Databases from Scoping Review). We recommended a distributive, procedural, and recognitional justice lens to FOCI as a way to inform future community engagement activities that will be used in climate planning given their utility when used in different rural and remote contexts. Documents were included based on three key factors: (1) utilizing a place-based context to climate change mitigation and adaptation, (2) inclusion of procedural, recognitional and distributive justice and (3) best practices for community participation in planning. In total, 33 climate adaptation resources met these criteria and were included and developed into a community engagement report. Phase 5: The findings from this review and insights from the aforementioned phases were then applied to the development of a Cortes Island survey for residents about climate change planning on the island. More specifically, the scoping review, community interviews, observations from the focus group, and environmental scan informed which questions were asked, the phrasing of the questions, and our dissemination strategy for the community survey [questions can be found in the Supplementary material (Draft Survey Questions Informed by Scoping Review)].

This paper brings together reflections from our team of researchers and community members on lessons learned from each step of the partnership and project development. Each phase of our project was iterative and led to the development of the next steps, clarified areas of focus, and aided in the development of deliverables. This paper is not intended to summarize in detail the findings from each of the five phases of work, but rather, to offer insights into the lessons learned from working together across the life course of this project and within a university-remote community partnership.

### 3.1 Framework

Rooting data science in theoretical frameworks, in our case in eco-social and critical theory, promotes the meaningful development of research programs which pay careful attention to the epistemological, ontological and empirical orientations central to the program of research.

In this study, we rooted our approach to research and partnership development in eco-social approaches to health, critical data studies and critical place inquiry. Eco-social approaches to health acknowledge “the reciprocity among the ecological and the social as essential features of a proactive orientation to future health and collective well-being, especially in the face of rapid planetary-scale ecological changes that threaten human well-being and societal stability” (21) (p. 61). Eco-social approaches to health highlight the role of relationships and interconnections between ecological and social systems (and challenge constructed dichotomies between the two) thus taking an orientation that pays homage to Indigenous knowledges and epistemologies (21). Using an eco-social approach to health also led us to engage with social and environmental determinants of health, with a specific interest in how the local level was reflected in the datasets. This material was included in the environmental scan, informed the development of the questions that were asked of community and the information that was deemed relevant in the asset mapping, and the posing of the justice question that guided the development of the community engagement report. This public health orientation to engaging in climate change planning, extends from calls from Parkes et al. (20) and Hancock (22) to utilize an eco-social approach in public health to address climate change.

Rooting our data science approaches in equity-driven and critical theoretical frameworks, such as critical data studies, acknowledges that data and how they are analyzed, are not neutral categories and processes (23–25). Rather, data also always represents people, places, structures and relationships and thus are contextual, relational, and situated in place-based settings (25, 26). Grounding data considerations in principles of critical place inquiry centers the need to develop research methods that account for meaningful considerations of both place and social positions (27). Data and science rooted in Western philosophy and capitalism bakes into its logic a dualism between humans and nature (28). To correct for these ontological biases, we have sought to ground our work in place—not conceived of as a static material context but rather as a living space, a place of social reproduction, ecological unfolding and a dynamic set of interrelationships that change over time and through the interplay between people, species, and social practices (27, 29, 30). Further, critical place inquiry urges us to notice how social locations and realities influence how people experience “place,” and also how they understand and influence these contexts (27). Place is not only a social construct, but also refers to the very land, water, air and mountains that constitute these places. Less seemingly permanent characteristics, such as flora, fauna and even weather patterns, also interrelated to produce a specific place at a particular time (27). With this lens, researchers must center (rather than keep peripheral or secondary) commitments to Indigenous sovereignty, the non-abstraction of nature, and the value of Indigenous epistemologies in relation to the fundamental ideas that land and water are life (27, 29).

One way that theory meets practice is in the very structuring of the research process, including the questions that we ask of ourselves and one another. Within the context of our work on community climate change adaptation and mitigation research, policy and practice, productive questions include: What counts as ‘data’? Who is represented and who is missing in existing data? Why do these gaps exist? How can we effectively integrate social, environmental and climate data to tell a story of interaction? Who has access to existing data and who has the knowledge to use them meaningfully? How do we bring in more-than-human needs and experiences to data? What do we need to enact to avoid using data in stigmatizing or exploitative ways? What are the limitations of standardized data when working with rural and remote communities and on eco-social phenomena? Who needs to be involved in the selection of relevant data? How far can the findings from data bring us without ground-truthing them in place and local experience? How can data be used to identify a community’s assets and strengths? In other words, how can we move away from treasuring what we can (easily) measure and rather learn to measure what we treasure?

Climate change, among many of the complex problems we face at the intersection of health and environment, are fundamentally equity issues (31–34). Given that climate change impacts—when assessed through a holistic lens—span social, health, political and economic sectors, it remains important to understand the intersections between climate change and social, environmental, ecological and planetary Determinants of Health (DoH) across scales of individual, community, and eco-social systems (4). Therefore, we argue that issues of climate change and environmental health benefit from grounding data collection, analysis, and reporting in critical place inquiry wherein place-based and disaggregated data can be produced and analyzed through reflexive and equity informed practices. These complex data are suited for the task of unpacking and concretely addressing the complex issues we are facing, including ‘wicked problems’ such as climate change and zoonotic pandemics which emerge out of contexts of social and environmental degradation. Additionally, it is crucial to link these phenomena with the structural and systemic layers of marginalization and oppression which exist. Therefore, when working on issues that intersect community and environmental health, we must acknowledge the impact that colonial and capitalist structures have had on our conceptualizations of ‘environment’, ‘community’ and ‘health’. A clear example being, how water is commonly talked about in policies and practices as a hazard, resource and access to it as a human right, rather than as an entity worthy of rights itself and as a source of life through which human societies are intertwined in networks of reciprocity, accountability and responsibility (35).

## 4 Results from the five phases

### 4.1 Phase 1: environmental scan

The results of our environmental scan illustrated that there were significant, and in some cases, irreconcilable differences between regional district and census data and the living realities of island residents. For example, existing data about Cortes Island prior to 2022 identified that less than 5% of homes on Cortes Island need repairs, 75% of the residents are homeowners, the majority of residents are food secure, and that roads are suitable for biking (16). However, through discussion between FOCI, the research team (many of whom are community members themselves) and scoping conversations with residents, we learned that these findings were not representative of lived experience or existing services and infrastructure. For example, a large number of Cortesians struggle annually with housing quality, stock availability and affordability along with serious issues of food security. Further, mobility is a chronic problem for many as there is little public transportation infrastructure, fuel prices are high and many roads on Cortes Island are unpaved, steep, windy, unlit, and have deep potholes, and many Cortes Island residents speak about serious crashes occurring on-island. The findings from this environmental scan and the processes of ground truthing data with locals emphasizes the importance of community engagement in research and decision-making processes to not only improve data accuracy, but to also increase the likelihood that research and data can accurately inform the development of more equitable and appropriate planning and climate change processes. This is particularly true for research regarding rural, remote, Northern and Indigenous communities as much of Canada’s existing climate change adaptation and mitigation efforts have been rooted within urban contexts, with a focus on infrastructure (i.e., public transportation electrification, mobility pricing, retrofitting) and larger scale populations that can be more easily characterized in standardized data gathering processes. Therefore, climate change planning must retain flexibility so efforts can be re-envisioned for applicability and equity in non-urban contexts, where infrastructure and key determinants of health such as public transportation, personal vehicles, food security, employment and housing are constrained.

However, despite its drawbacks and various data limitations, the Environmental Scan did illustrate various strengths and initiatives taking place on Cortes Island. Working in community contexts, it is important to acknowledge and name the agency, assets and activism of communities. For example, despite the aforementioned challenges facing Cortes, the Klahoose First Nation’s Indigenous environmental leadership and actions include a range of initiatives, such as the development of a clean energy project, the investment of generators to protect local food harvests during power outages and the development of bivalve shellfish aquaculture as a key development of low-carbon futures, income, and food security. These initiatives center the vast Traditional Ecological Knowledge, which is not held by settler communities and help to build a future that is grounded in the communities, values, priorities and commitments to flourishing (16). Centering Indigenous voices and leadership in climate change adaptation and mitigation efforts, and drawing connections between the health of ecosystems and health of communities, is vital not only for Truth and Reconciliation but also for building sustainability, restoration and wellbeing into climate planning.

### 4.2 Phase 2: drought report, heat-pump resources and community asset report

Once it became clear the Environmental Scan of academic and gray literature did not provide an adequate understanding of social and ecological conditions on Cortes Island, the research team worked closely with FOCI to understand current issues facing the community related to climate change, education, and outreach. Themes of importance included the impacts of the Fall 2022 drought experienced on the island, energy and efficient heating (14) and mobilizing knowledge about existing eco-social assets such as the Children’s Forest and the work of the Social Profit sector (17).

The next steps taken by partnership were to conduct five informal scoping interviews with community members about their knowledge and expertise related to the assets on the island that were not represented in the Environmental Scan. Cortes Island serves as a generative example of the power of diverse knowledges and experiences and illustrates how these knowledges can provide nuance and context to quantitative data. For example, the sister nations the Klahoose, the Tla’amin, and Homalco Nations hold knowledge built from living on their traditional territories for thousands of years. Knowledge is also shared from those whose knowledge has been built through generations of place based living on Cortes Island, from those across the life course—from children, young families, through to seniors and elders, and from professionals and academics, including marine biologists, mycologists, botanists, ecologists, geologists and wetland restoration experts. Children and youth growing up on the island also have a unique set of important insights to share. For example, wetlands and old growth rainforest ecosystems emerged as eco-social assets from our informal interviews (17). Local knowledge shared was gained through observations of environmental complexity derived from engaging with the system holistically, through relational heuristics and living experiences (17, 36). People shared information about local disaster risk management strategies, insights from the decades of data they collected in physical notebooks while monitoring salmon streams, and examples of the co-benefits of ecosystem restoration and wildfire management due to the Island’s wetland restoration work.

Through these diverse epistemic lenses, the community helped us ground-truth existing data and begin to identify ways to fill gaps where data are missing. Relational heuristics, those localized observations made over time which are often transmitted through narrative stories or descriptions were also crucial. Thus, including a range of knowledge holders in data and knowledge production can address gaps in baseline data and place decontextualized knowledge back into place are two strategies that have helped to address significant impediments that had initially impacted the development of the Cortes Island resilience plan. By giving locals a chance to engage with local initiatives there is a greater likelihood that research and projects ‘about and with’ a community can be harnessed to create more meaningful change. As such, it is a disservice to view community engagement acts - such as consultation and learning from local and traditional ecological knowledge - as simply a matter of ethical behavior and responsibility. Rather, viewing the community as critical to research, we improve the integrity, accuracy, and utility of our data and increase research capacity to meaningfully and adequately address data gaps and community needs.

### 4.3 Phases 3–5: justice-informed community climate planning report and the social profit forum

After the environmental scan and community asset mapping process, our team conducted a scoping review of the literature describing community inclusion in local climate planning and shared our findings in a community report.

Our scoping review of the literature explored how, respectively, distributive, procedural and recognitional justice provide an approach to community engagement that retains attention to the envisioning and building of a collective future (37, 38). Distributive justice is concerned with how social, economic, and ecological goods are distributed as well as the impacts of climate change (37, 38). Participatory justice is concerned with who is included in the decision-making processes and how they are included in terms of power dynamics and role. Recognitional justice is concerned with who is acknowledged in terms of the impacts and often references intersectional identities (e.g., socioeconomic status, culture, gender identity) (37, 38). Therefore, in thinking about and actioning climate justice at the local level, engaging community members has been recognized as a robust starting point for cultivating climate justice (39).

Literature also highlights that local knowledge and a sense of place form the basis for meaningful community engagement within climate change planning processes. For example, Johnson et al. (39) state “notions of place attachment, sense of place, the role of culture, and sense-making in social discourse are increasingly being used to understand the complex interactions between society and the environment… and how societies respond and adapt to change” (40). The research also reminds that the impacts of climate change are felt across all scales, but at the local level the impacts will be unique to each community; therefore, it is important to consider how external drivers may affect the existence, sustainability and allocation of resources to the community (40). Community perspectives were sought in a variety of ways. For example, during the development of the community engagement report, a team member attended the Social Profit Forum where it was highlighted by the Social Profit Network (a network of social profits and nonprofits on Cortes), that community members on Cortes “face higher than average costs and lack of access to basic needs such as housing, transportation, healthy food, mental health support, education, laundry facilities, private childcare and insurance as compared to the average BC resident” (19, 41). Attending this gathering of local social service providers confirmed many of the inaccuracies in the Environmental Scan, and informed the development of a new suite of future community engagements activities that were presented to FOCI. The value of the capacity of the Social Profit Sector to convene cross cutting conversations on issues such as housing, climate change and poverty was clear as was their attention to power, representation and voice, for example by making conscious efforts to reflect on who is and is not in the room. To support this equity commitment, we shared recommendations from our review of community engagement literature which advocated for providing multiple mediums for engagement, including meeting in person and online and providing multiple mediums of information exchange, such as via surveys, meetings and gatherings. In a small remote community like Cortes Island, directly inviting members of the community, such as youth and the Klahoose First Nation, to participate is important in ensuring equitable participation. For complex topics such as climate change, it is important that processes are stewarded through strong facilitation which is rooted in an awareness of human and more than human health and wellbeing, cultural humility, solidarity, power and privilege, intergenerational inequalities, trauma- and healing-informed approaches and structural forms of marginalization (41, 42).

Our community report also recommended FOCI pursue scenario planning as a potential future activity as this method of community engagement is increasingly being used to create adaptation strategies for climate change that center justice (10, 40, 43). Scenario planning involves descriptions of various scenarios that have the potential to unfold within specific spaces, places and times and draw upon available science to promote decision making (40, 43). Scenario planning is particularly useful in exploring uncertainty as it allows for the integration of knowledge, interests and opinions as well as creates a process of community learning and dialogue with diverse community members (40). In addition to integrating elements of uncertainty, scenario planning allows for the integration of present and future assumptions and invites community members to come together to envision a range of collective futures (10) and to consider how to build pathways to these futures.

The findings from the community report were then drawn upon by FOCI and research team members to develop a Cortes Island survey to be administered to residents about climate change needs and priorities on the island. More specifically, this review informed which questions were asked, the phrasing of the questions, and our survey dissemination strategies. Questions were developed to reflect the value and goals of justice-oriented community climate planning and thus asked questions about: “*For what?*: How do we ground our thinking about the boundaries of systems that affect our lives in these types of processes?”; “*To what?*: How do we think about what the socio-ecological systems that we interact with are impacted by?”; “*For who?*: Who will our solutions benefit? What community assets exist that could be leveraged to build resilience to different climate futures?”; and “*Over what time frame?*: What timescale are we considering in our planning processes?”

## 5 Discussion

This project, conducted in part by ‘insider/outsider’ scholars, climate scholars, community organizers, and activists, draws lessons from engaging in a community-based climate planning process in a remote island community context and highlights the complexity of developing community climate plans that address data gaps, attend to social inequities and seek to build concrete climate action. This work also highlights tensions that arise when most data are deficit-based yet the literature advocates for centering the needs of the community, being responsive to place-based contexts and nuanced in an understanding of how health and wellbeing is produced in the nexus of social-ecological interactions. In this research, however, these core research insights dovetailed with community knowledges where residents drew from their understanding and experiences of living on Cortes Island where the social, economic and ecological experiences of living remotely and experiencing climate change on an island home are often directly felt. Thus, in alignment with existing literature, it is clear that through the experiences of community members we can understand local impacts of climate change, identify adaptive capacity and articulate how these intersect with local and regional contexts (41) and address issues of inequity arising within socio-ecological systems interactions (10). In acknowledging how social, economic and ecological systems are connected, we can then work to consider how future changes and adaptations may affect individuals within a community. Therefore, thinking at the local level about systems allows us to recognize links between different issues, which allows for co-benefits planning that seeks to address multiple community challenges (42).

Historically, top-down approaches to climate change mitigation and adaptation have created a stark division between citizens, science and policymakers, which have led to largely ineffective actions and limited buy-in (39). Without effective community engagement, power dynamics can define the boundaries of the socio-ecological systems in ways that serve a minority of perspectives and lead to inequitable solutions to community challenges (10). As is evident both in the available literature and our project’s findings, acknowledging the value of local and traditional ecological knowledge is critical to supporting equity through data as the qualitative observations that inform them allow us to paint a fuller picture of health in the interplay between social, ecological and climatic processes, while also accounting for the cultural importance of species and places. Failing to do so runs the risk of using communities as a source for data extraction; studying them to further academic research without also addressing community concerns and taking local knowledge seriously, as well as running the risk of producing data which are erroneous, incomplete or biased. Community knowledge can also be empirically important, for instance, there is a lack of scientific literature on salmon spawning runs on Cortes Island; however, locals routinely collect data on streams and salmon-return data that may not be shared with/of interest to policy makers and government (e.g., the Friends of Cortes Island Society streamkeepers) (17).

Multiple members of our team hold insider-outsider roles as researchers, as community members and activists. As a part of our reflective practice on this project, we discussed the tensions that exist between community-relevant outputs and the types of work and ways of working that are valued by the scientific/academic community. Acknowledging the limitations and colonial structures which guide academic practices, our team engaged in ongoing reflection to ensure that the priorities of the communities remained central in this project. To put academic processes in service to community, we identified what skills and assets we have available to provide to community organizations, such as access to paywalled literature and experience conducting academic literature reviews and reports. For the researchers on our team, we often found ourselves questioning how to be in service to academia and our community at the same time. Thinking about the utility of our outputs and the contribution of the data we collected through academic literature, gray literature and informal interviews with the community was a humbling process for the early career researchers on our team who are also grappling with the complexity of both wicked issues such as climate change and how academia can be in service to community organizing and planning processes. Prioritizing integrated knowledge mobilization strategies was an important element of this work as well. As illustrated by the Canadian Institute for Health Research, “the central premise of iKT is that involving knowledge users as equal partners alongside researchers will lead to research that is more relevant to, and more likely to be useful to, the knowledge users” (44). Integrated knowledge translation calls for reflexive, ethical co-production of knowledge in academic spaces rooted in a critical analysis of power dynamics, which is a process members of the team discussed at every stage of the project and led to FOCI leading the informal interviews and holding the data as well as outputs oriented toward the community (45).

This project effectively remained iterative and responsive to community interests and needs, and showcased strategies for doing place-based work on climate action planning. Despite these strengths, this research has some limitations, including being less applicable to other settings given how focused our methods were on this particular community context, working with a small group of community organizations to set the foundational work for climate planning on the island, and moving beyond the bounds of what some may argue is the purview of classical climate resilience planning given the feedback loops between a range of intersecting structural issues, from housing and food insecurity to poverty and lack of community infrastructure.

## 6 Conclusion

In working toward equitable data approaches that enable us to measure what we treasure, we underscore the value of mixed-methods approaches such as the inclusion of community knowledges when working with quantitative data in order to uplift multiple ways of knowing and to meaningfully center these rich qualitative data in climate adaptation and mitigation planning. Our work with the Cortes Island community has highlighted the complexities of what counts as data, identified issues of quantitative data limitations and inaccuracies, and raised issues of how to increase justice-oriented community involvement. In developing the type of integrative solution-oriented thinking we need to address issues of climate change and community health, we found that the following is required: (1) collaborating across sectors and disciplines to measure, evaluate and monitor social, environmental, climate and health data; (2) building in mechanisms of researcher accountability to communities through the ground truthing of data with the people that these findings are about; (3) using stories of community challenges and successes to guide research; and, (4) revealing and advocating for filling silences in data collection by leveraging mixed-methods and integrating epistemic diversity into knowledge formation processes.

The immediacy of climate change mitigation and adaptation should not abdicate our responsibilities as researchers to address the inequities inherent in our systems of data collection, integration and analysis, which themselves are enmeshed in the colonial and capitalist epistemologies and structures that concurrently work to perpetuate and deepen the climate crisis. Our responsibilities extend beyond our human relations to the ecosystems within which our individual and community health is embedded and thus lead us to support taking a more than human approach to climate planning. This entails going upstream until it becomes clear that the ecosystems are our health systems, advocating to dismantle the silos within which we address community health and promoting an embrace of interventions that aspire to identifying co-benefits and engage in multisolving (35). We suggest that when applying the findings from this work to other communities’ climate adaptation and mitigation planning processes, that the foci, phases, and processes be tailored to the specific social, ecological, and place-based contexts and community needs, assets, and interests of each place because while climate change is a global phenomenon it is experienced and responded to in the intimacy of our own lives, homes, neighborhoods and communities.

## Data availability statement

The original contributions presented in the study are included in the article/Supplementary material, further inquiries can be directed to the corresponding author.

## Author contributions

AK: Conceptualization, Data curation, Formal analysis, Funding acquisition, Methodology, Project administration, Writing – original draft, Writing – review & editing. KT: Conceptualization, Data curation, Formal analysis, Methodology, Validation, Writing – original draft, Writing - review & editing. FB-H: Data curation, Formal analysis, Methodology, Validation, Writing – original draft, Writing – review & editing. SC: Data curation, Formal analysis, Writing – review & editing. MT: Conceptualization, Supervision, Validation, Writing – review & editing. MAG: Validation, Writing – review & editing. MKG: Conceptualization, Funding acquisition, Methodology, Supervision, Writing – original draft, Writing – review & editing.
