# Supplementary material for: Promoting community health and climate justice co-benefits: insights from a rural and remote island climate planning process
Source: Front Public Health. 2024 Mar 12;12:1309186. doi: 10.3389/fpubh.2024.1309186 (PMC10964719; doi:10.3389/fpubh.2024.1309186)
Supplement: Supplementary file 1 [file Data_Sheet_1.PDF]

Supplementary Materials: Datasets Identified in Environmental Scan

The first step in the creation of the environmental scan and gap analysis was the creation of an annotated bibliography identifying adaptation-related documents for Vancouver Island and BC coastal communities. During this process, the Climate Adaptation Environmental Scan and Gap Analysis prepared for Metro Vancouver was identified as a strong resource to support the development of CCA planning, and was thus used as a model for the present document. With a shared purpose, this document provided a methodological roadmap as well as themes and areas of interest to consider. Additionally, the Salt Spring Island Climate Action Plan (CAP) was identified as highly congruent with the Cortes Island context, providing a superb source of inspiration and guidance regarding targets, goals, and climate change themes to consider. Beyond these two key reports, documents were included in this report based on three key factors: (1) context congruency with Cortes Island, (2) inclusion of comprehensive implementation/monitoring and evaluation recommendations, and (3) integration of the eco-social (or an equivalent) approach to climate health. In total, 22 climate adaptation resources that met these criteria were included. This included 4 climate adaptation strategies/plans, 2 publications to help inform climate strategizing and planning, and 16 resources to help guide data collection, seek funding and partnerships, and guide the creation and implementation of a CAP."

1. Cortes Island: A small island rich with stories. Campbell River Mirror. 2018 May 24;
2. Melanie Kurrein, Crystal Li, Drona Rasali, Henry Lau. Food Costing in BC 2017: Assessing the affordability of healthy eating [Internet]. Provincial Health Services Authority; 2018. Available from: http://www.bccdc.ca/pop-public-health/Documents/food-costing-BC-2017.pdf
3. Statistics Canada. Census profile, Strathcona B, Regional district electoral area [Internet]. 2016. Available from: https://www12.statcan.gc.ca/census-recensement/2016/dp-pd/prof/details/page.cfm?Lang=E&Geo1=CSD&Code1=5924052&Geo2=CD&Code2=5924&Data=Count&SearchText=strathcona&SearchType=Begins&SearchPR=01&B1=All&TABID=1
4. Cortes Island Economic Plan Survey [Internet]. Cortes Island Business and Tourism Association; 2017. Available from: http://cibata.ca/wp-content/uploads/2018/01/Cortes_Island_LEAP_Results_Oct_2017-Condensed-1.pdf
5. Government of Canada. Who is most impacted by climate change [Internet]. 2022. Available from: https://www.canada.ca/en/health-canada/services/climate-change-health/populations-risk.html
6. Klahoose. Klahoose First Nations [Internet]. Available from: https://www.klahoose.org/about
7. Rochelle Baker. Small coastal First Nation fuels economic growth with clean energy. Canada's National Observer [Internet]. 2022 Feb 14; Available from: https://www.nationalobserver.com/2022/02/14/news/small-coastal-first-nation-fuels-economic-growth-clean-energy
8. Nazrul Islam, John Winkel. Climate Change and Social Inequality. Dep Econ Soc Aff [Internet]. 2017;152. Available from: https://www.un.org/esa/desa/papers/2017/wp152_2017.pdf
9. school district 72. Human early learning partnership, early development instrument [Internet]. 2014. Available from: www.earlylearning.ubc.ca
10. Strathcona Community Health Network. Social determinants of health fact sheet: Cortes Island [Internet]. Available from: https://srd.ca/wp-content/uploads/2019/06/Cortes-Community-Profile.pdf

11. Climate adaptation environmental scan and gap analysis prepared for Metro Vancouver [Internet]. The Arlington group planning and architecture; 2015. Available from: http://www.metrovancouver.org/services/air-quality/AirQualityPublications/ClimateChangeAdaptationScanandGapAnalysis.pdf
12. Transition SaltSpring. Salt Spring Island climate action plan [Internet]. 2021. Available from: https://transitionsaltspring.com/wp-content/uploads/2021/01/1.-CAP-2.0-COMPLETE-WEB.pdf
13. BC climate change adaptation program [Internet]. 2021. Available from: https://bcclimatechangeadaptation.ca/
14. Krawchenko, T, Rhodes, K, Harrison, K, Pearce, K, Shaw, K, Brousselle, A, et al. Territorial analysis and survey of local government priorities for climate action: Vancouver Island and coastal communities [Internet]. Vancouver Island and Coastal Communities Climate Leadership Plan, Victoria, B.C.; 2020. Available from: https://pics.uvic.ca/sites/default/files/vicc_report_highlights_final_formatted.pdf
15. Charlotte K. Whitney, Tugce Conger, Natalie C. Ban, Romney McPhie. Synthesizing and communicating climate change impacts to inform coastal adaptation planning. FACETS [Internet]. 2020;5(1). Available from: https://doi.org/10.1139/facets-2019-0027
16. British Columbia Assembly of First Nations. BC First Nations climate strategy and aciton plan development progress update [Internet]. Available from: https://www.bcafn.ca/climate-emergency/bc-first-nations-climate-strategy-and-action-plan-development-progress-update#:~:text=The%20objective%20of%20the%20Strategy,resilience%20in%20First%20Nation%20communities.
17. Cheyenne Arnold-Cunningham, Josh Kioke, Patricia Rojas. BC First Nations climate strategy and action plan [Internet]. 2021 Mar. Available from: https://www.bcafn.ca/sites/default/files/docs/reports-presentations/BCFNCSAP%20-%20BCAFN%20Presentation%20(March%204%202021).pdf
18. British Columbia, clean BC. Climate preparedness and adapation strategy - Draft strategy and phase 1 actions for 2021-2022 [Internet]. 2021. Available from: https://www2.gov.bc.ca/assets/gov/environment/climate-change/adaptation/cpas_2021.pdf#page=45
19. Adapting to climate change on the British Columbia Coast [Internet]. Available from: https://ucluelet.ca/images/Adapting_to_Climate_Change_on_the_BC_Coast.pdf
20. Climate Action Planning Guide [Internet]. Climate Smart Communities; Available from: https://cdrpc.org/wp-content/uploads/2015/05/CAP-Guide_MAR-2014_FINAL.pdf
21. Climate ADAPT - Sharing adapation knowledge for a climate-resilient Europe [Internet]. Available from: https://climate-adapt.eea.europa.eu/eu-adaptation-policy/sector-policies/coastal-areas/index_html/#policy-framework
22. Wang T, Hamann A, Spittlehouse D, Carroll C. ClimateBC [Internet]. 2016. Available from: https://climatebc.ca/
23. Rochelle Baker. Coastal First Nation gets a taste of success with seaweed. Canada's National Observer [Internet]. 2021 May 17; Available from: https://www.nationalobserver.com/2021/05/17/news/coastal-first-nation-gets-taste-success-seaweed
24. Community-driven climate resilience planning: A framework [Internet]. National Association of Climate Resilience Planners; Available from: https://kresge.org/sites/default/files/library/community_drive_resilience_planning_from_movement_strategy_center.pdf

25. Union of BC municipalities. Community emergency preparedness fund [Internet]. 2022. Available from: https://www.ubcm.ca/cepf
26. Forest enhancement society of British Columbia [Internet]. Available from: https://www.fesbc.ca/
27. British Columbia. How B.C. is preparing for climate change [Internet]. Available from: https://www2.gov.bc.ca/gov/content/environment/climate-change/adaptation/preparing-actions
28. First Nations Health Authority. Indigenous Climate Health Action Program [Internet]. 2021. Available from: https://www.fnha.ca/what-we-do/environmental-health/climate-health-action-program/project-snapshots-2021
29. Klahoose Wilderness Resort [Internet]. Available from: https://www.klahooseresort.com/
30. BC Parks Living Lab. Living lab for climate change and conservation [Internet]. Available from: https://bcparks.ca/partnerships/living-labs/
31. Marine Planning Partnership Initiative [Internet]. 2015. Available from: http://mappocean.org/wp-content/uploads/2015/11/MarinePlan_NorthVancouverIsland_28072015_corrected.pdf
32. Pacific Climate Impacts Consortium, University of Victoria. Plan2Adapt [Internet]. Available from: https://www.pacificclimate.org/analysis-tools/plan2adapt
33. Pacific Institute for Climate Solutions [Internet]. 2012. Available from: https://pics.uvic.ca/
34. Government of Canada. Pan-Canadian Framework on Clean Growth and Climate Change [Internet]. 2018 Jan. Available from: https://www.canada.ca/en/services/environment/weather/climatechange/pan-canadian-framework/climate-change-plan.html
35. Abate RS, Elizabeth Ann Kronk. Chapter 1: Commonality among unique indigenous communities: an introduction to climate change and its impacts on indigenous peoples [Internet]. 2013. Available from: https://www.elgaronline.com/view/edcoll/9781781001790/9781781001790.00009.xml

Supplementary Materials: PRISMA Chart of Scoping Review

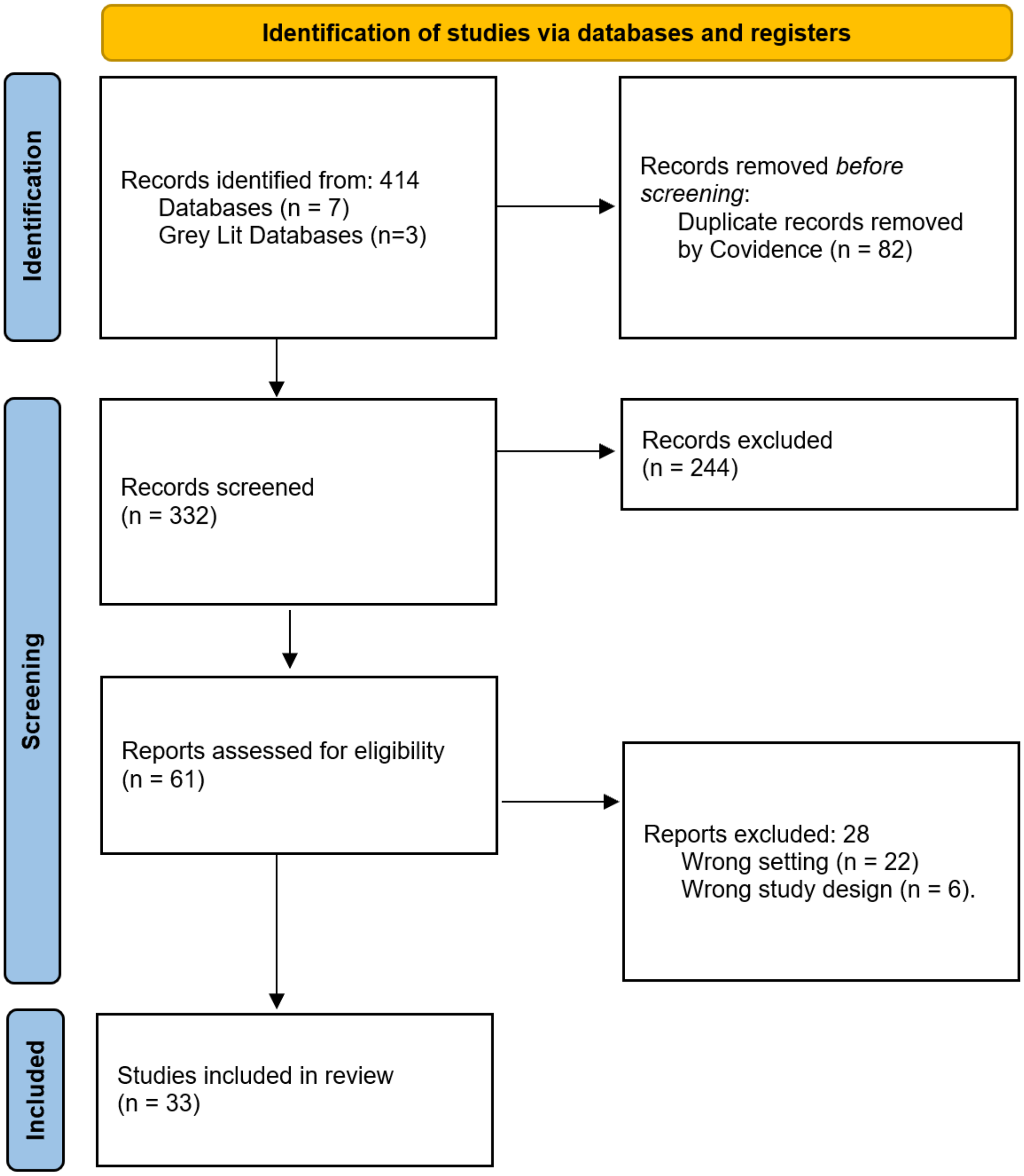

Supplementary Materials: Search Strategy, Terms, and Databases from Scoping Review

MeSH:
A (climate change): Climate change OR global warming OR climatic processes OR greenhouse effect OR Climate OR sustainable development
B (planning): social planning OR city planning OR environment design OR universal design OR "risk evaluation and mitigation" OR community-institutional relations OR interinstitutional relations OR conservation of natural resources/methods OR conservation of natural resources/legislation & jurisprudence OR conservation of natural resources/trends
C (Community inclusion/participation/leadership): [Community participation/legislation & jurisprudence OR community participation/statistics & numerical data OR community participation/trends] OR stakeholder participation
D (justice): environmental justice OR diversity, equity, inclusion OR social change

Keywords:
A (climate change): "climate crisis"
B (planning): "community involvement" OR "climate adaptation" OR "climate mitigation" OR coping OR cope
C (Community inclusion/participation/leadership): "community leadership" OR "community inclusion" OR "participation"
D (justice): "distributive justice" OR "procedural justice" OR "recognitional justice" OR justice

Databases:

Academic
- Environment Complete
- EBSCO
- Political Science Complete
- BioOne
- Scopus
- Web of Science
- GEOBASE

Grey
- EcoCat The Ecological Reports Catalogue from the BC Ministry of the Environment
- Government of Canada Publications
- Google

Environment Complete
"(Climate change OR global warming OR climatic processes OR greenhouse effect OR Climate OR sustainable development OR "climate crisis") AND (social planning OR city planning OR environment design OR universal design OR "risk evaluation and mitigation" OR community-institutional relations OR interinstitutional relations OR conservation of natural resources/methods OR conservation of natural resources/legislation & jurisprudence OR conservation of natural resources/trends OR "community involvement" OR "climate adaptation" OR "climate mitigation" OR coping OR cope) AND ([Community participation/legislation &

jurisprudence OR community participation/statistics & numerical data OR community participation/trends] OR stakeholder participation OR “community leadership” OR “community inclusion” OR “participation”) AND (environmental justice OR diversity, equity, inclusion OR social change OR “distributive justice” OR “procedural justice” OR “recognitional justice”) Published Date: 20150101-20230531 on 2023-05-04 10:18 PM"
= 17 results

BioOne
ABSTRACT:(Climate change OR global warming OR climatic processes OR greenhouse effect OR Climate OR sustainable development OR "climate crisis") AND (social planning OR city planning OR environment design OR universal design OR “risk evaluation and mitigation” OR community-institutional relations OR interinstitutional relations OR conservation of natural resources/methods OR conservation of natural resources/legislation & jurisprudence OR conservation of natural resources/trends OR “community involvement” OR “climate adaptation” OR “climate mitigation” OR coping OR cope) AND ([Community participation/legislation & jurisprudence OR community participation/statistics & numerical data OR community participation/trends] OR stakeholder participation OR “community leadership” OR “community inclusion” OR “participation”) AND (environmental justice OR diversity, equity, inclusion OR social change OR “distributive justice” OR “procedural justice” OR “recognitional justice”), 2015, 2023
= 39 results

Political Science Complete

(Climate change OR global warming OR climatic processes OR greenhouse effect OR Climate OR sustainable development OR "climate crisis") AND (social planning OR city planning OR environment design OR universal design OR “risk evaluation and mitigation” OR community-institutional relations OR interinstitutional relations OR conservation of natural resources/methods OR conservation of natural resources/legislation & jurisprudence OR conservation of natural resources/trends OR “community involvement” OR “climate adaptation” OR “climate mitigation” OR coping OR cope) AND (Community participation OR stakeholder participation OR “community leadership” OR “community inclusion” OR “participation”) AND (environmental justice OR diversity, equity, inclusion OR social change OR “distributive justice” OR “procedural justice” OR “recognitional justice” OR justice)	Limiters - Publication Date: 20150101-20230531 Expanders - Apply related words Search modes - Boolean/Phrase

= 4 results

Scopus

ALL(Climate change OR global warming OR climatic processes OR greenhouse effect OR Climate OR sustainable development OR "climate crisis") AND (social planning OR city planning OR environment design OR universal design OR "risk evaluation and mitigation" OR

community-institutional relations OR interinstitutional relations OR conservation of natural resources/methods OR conservation of natural resources/legislation & jurisprudence OR conservation of natural resources/trends OR "community involvement" OR "climate adaptation" OR "climate mitigation" OR coping OR cope) AND (Community participation OR stakeholder participation OR "community leadership" OR "community inclusion" OR "participation") AND (environmental justice OR diversity, equity, inclusion OR social change OR "distributive justice" OR "procedural justice" OR "recognitional justice" OR justice) AND (LIMIT-TO (PUBYEAR,2023) OR LIMIT-TO (PUBYEAR,2022) OR LIMIT-TO (PUBYEAR,2021) OR LIMIT-TO (PUBYEAR,2020) OR LIMIT-TO (PUBYEAR,2019) OR LIMIT-TO (PUBYEAR,2018) OR LIMIT-TO (PUBYEAR,2017) OR LIMIT-TO (PUBYEAR,2016) OR LIMIT-TO (PUBYEAR,2015))

= 85 results

EBSCO

(Climate change OR global warming OR climatic processes OR greenhouse effect OR Climate OR sustainable development OR "climate crisis") AND (social planning OR city planning OR environment design OR universal design OR "risk evaluation and mitigation" OR community-institutional relations OR interinstitutional relations OR conservation of natural resources/methods OR conservation of natural resources/legislation & jurisprudence OR conservation of natural resources/trends OR "community involvement" OR "climate adaptation" OR "climate mitigation" OR coping OR cope) AND (Community participation OR stakeholder participation OR "community leadership" OR "community inclusion" OR "participation") AND (environmental justice OR diversity, equity, inclusion OR social change OR "distributive justice" OR "procedural justice" OR "recognitional justice" OR justice) Show Less	Limiters - Date Published: 20150101-20231231 Expanders - Apply equivalent subjects Search modes - Boolean/Phrase

- 12 results

Web of Science

(((AB=(Climate change OR global warming OR climatic processes OR greenhouse effect OR Climate OR sustainable development OR "climate crisis")) AND AB=(social planning OR city planning OR environment design OR universal design OR “risk evaluation and mitigation” OR community-institutional relations OR interinstitutional relations OR conservation of natural resources/methods OR conservation of natural resources/legislation & jurisprudence OR conservation of natural resources/trends OR “community involvement” OR “climate adaptation” OR “climate mitigation” OR coping OR cope)) AND AB=([Community participation/legislation & jurisprudence OR community participation/statistics & numerical data OR community participation/trends] OR stakeholder participation OR “community leadership” OR “community inclusion” OR “participation”)) AND AB=(environmental justice OR diversity, equity, inclusion OR social change OR “distributive justice” OR “procedural justice” OR “recognitional justice”) 2015, 2023

=288 results abstract screening

GeoBase

GEOBASE for 2015-2023: (((((Climate change OR global warming OR climatic processes OR greenhouse effect OR Climate OR sustainable development OR "climate crisis") WN KY) AND ((social planning OR city planning OR environment design OR universal design OR "risk evaluation and mitigation" OR community-institutional relations OR interinstitutional relations OR conservation of natural resources/methods OR conservation of natural resources/legislation & jurisprudence OR conservation of natural resources/trends OR "community involvement" OR "climate adaptation" OR "climate mitigation" OR coping OR cope) WN KY)) AND (([Community participation/legislation & jurisprudence OR community participation/statistics & numerical data OR community participation/trends] OR stakeholder participation OR "community leadership" OR "community inclusion" OR "participation") WN KY)) AND ((environmental justice OR diversity, equity, inclusion OR social change OR "distributive justice" OR "procedural justice" OR "recognitional justice") WN KY)) AND (English WN LA)
= 357 (subject, title, abstract)

Supplementary Materials: Draft Survey Questions Informed by Scoping Review

1. How long have you lived on Cortes?
2. How worried are you about climate change?
    1. Extremely worried
    2. Very worried
    3. Somewhat
    4. Not at all

7. How often do you talk to your friends and family about climate change?
    1. All the time
    2. Often
    3. Occasionally
    4. Never

8. Do you feel that climate change has affected you personally?
    1. Yes
    2. No

9. Do you vote based on climate change?
    1. No
    2. Yes

10. Do you invest based on climate change?
    1. No
    2. Yes

11. Do you shop based on climate change?
    1. No

2. Yes

12. Do you consider climate change when shopping for food?

    1. Yes
    2. Sometimes
    3. No

13. Do you grow your own food?

    1. Large farm
    2. Small farm
    3. Community garden plot
    4. Garden
    5. Pots/planters
    6. No

14. What are the barriers for you buying more food locally?

    1. Kinds of food available
    2. Cost
    3. Year round availability
    4. Time/ready-made foods

15. Would you consider changing your family's diet by doing any of the following?

    1. Vegan
    2. Vegetarian
    3. Already vegan
    4. Already vegetarian
    5. Meatless and dairy free three days a week

6. Meatless and dairy free one day a week

16. What are the barriers for you to grow more of your own food and increase local production?

1. Land access
2. Adequate sunshine
3. Deer
4. Time
5. Sufficient water
6. knowledge/experience
7. Other

17. Do you have any of the following?

1. Rain barrels
2. Greywater system
3. Cistern
4. Well

18. Do you have a shallow or a deep well?

1. Shallow
2. Deep
3. N/A

19. Do you ever find you run out of water?

1. Yes
2. No

20. In the event of a drought, would you consider sharing your water with the community?

1. Yes
2. No

21. What kind of home do you live in?

    1. Vacation home
    2. Looking for housing
    3. Tiny home
    4. Duplex/Multi-plex
    5. Detached single family home
    6. Mobile home

22. What are your barriers to buying an EV?

    1. Other
    2. Availability of charging stations
    3. Time to recharge
    4. Cost
    5. Range anxiety

23. What is your main mode of transportation currently?

    1. SUV
    2. Truck
    3. Car
    4. Biking
    5. Walking

24. Would you consider buying an electric vehicle as your next vehicle?

    1. Already drive an EV

2. Maybe
3. No
4. Yes

25. How much do you compost?

1. Compost at home and use the soil in my garden
2. Most of the time
3. Sometimes
4. Never

26. What climate events have you witnessed on Cortes Island?

1. High rain or snowfall, above what you would consider normal
2. Increased severe storm event
3. Flood
4. Invasive species
5. Severe cold periods
6. High temperatures for a prolonged period of time
7. Other severe weather-related event
8. Drought
9. Climate related change to vegetation
10. Climate-related change to animal populations
11. Human health-related
12. Fire
13. Other (please specify)

27. Please rank which climate events concern you most on Cortes Island

1. High rain or snowfall, above what you would consider normal
2. Increased severe storm event
3. Flood
4. Invasive species
5. Severe cold periods
6. High temperatures for a prolonged period of time
7. Other severe weather-related event
8. Drought
9. Climate related change to vegetation
10. Climate-related change to animal populations
11. Human health-related
12. Fire
13. Other (please specify)

28. Share how you have seen Cortesians respond to any of the following

1. High rain or snowfall, above what you would consider normal
2. Increased severe storm events
3. Flood
4. Invasive species
5. Severe cold periods
6. High temperatures for a prolonged period of time
7. Other severe weather-related event
8. Drought
9. Climate related change to vegetation

10. Climate-related change to animal populations

11. Human health-related

12. Fire

13. Other (please specify)

29. Share what community assets exist that could be leveraged to build community resilience and respond to these stresses

30. What existing vulnerabilities (i.e lack of housing, food insecurity) do you see as being made worse by climate change?

31. Have you engaged in fireproofing your home/property? If so, what have you done?

32. Do you believe you will be impacted by sea-level rise?

33. How much comfort/leisure would you be willing to forgo in order to ensure others have their basic needs met?

34. Do you think about where your food comes from? How do you think you will be impacted by disruptions to global food supply chains (driven by climate induced drought, flooding, conflict etc)?

35. Climate change is an equity issue. Many of the people who have contributed to the least to climate change will be the most affected. How do you think we should share the burden of mitigating our greenhouse gas emissions and adapting to climate change given the equity dimensions of this issue?

36. What skills/resources do you have to contribute to responding to climate change events and building community resilience to climate change?

37. If you are a landowner, would you be willing to share your land/resources to promote community resilience? What would this look like for you?

38. What community organizations are you a part of, and how do you think your organization will be affected by climate change?
